# Estimating Meningitis Hospitalization Rates for Sentinel Hospitals Conducting Invasive Bacterial Vaccine-Preventable Diseases Surveillance

**Published:** 2013-10-04

**Authors:** Jason Mwenda Mathiu, Pushpa Ranjan Wijesinghe, Mary Agócs, Anthony Burton, James Sale, Chris Van Beneden, Thomas Taylor, Brendan Flannery

**Affiliations:** World Health Organization Regional Office for Africa, Brazzaville, Congo; World Health Organization Regional Office for Southeast Asia, New Delhi, India; Dept of Immunizations, Vaccines, and Biologicals, World Health Organization, Geneva, Switzerland; Div of Bacterial Diseases, National Center for Immunization and Respiratory Diseases; Global Immunization Div, Center for Global Health, CDC

The World Health Organization (WHO)–coordinated Global Invasive Bacterial Vaccine-Preventable Diseases (IB-VPD) sentinel hospital surveillance network provides data for decision making regarding use of pneumococcal conjugate vaccine and *Haemophilus influenzae* type b (Hib) vaccine, both recommended for inclusion in routine childhood immunization programs worldwide (1,2). WHO recommends that countries conduct sentinel hospital surveillance for meningitis among children aged <5 years, including collection of cerebrospinal fluid (CSF) for laboratory detection of bacterial etiologies (3). Surveillance for pneumonia and sepsis are recommended at selected hospitals with well-functioning laboratories where meningitis surveillance consistently meets process indicators (e.g., surveillance performance indicators) (3). To use sentinel hospital surveillance for meningitis to estimate meningitis hospitalization rates, WHO developed a rapid method to estimate the number of children at-risk for meningitis in a sentinel hospital catchment area. Monitoring changes in denominators over time using consistent methods is essential for interpreting changes in sentinel surveillance incidence data and for assessing the effect of vaccine introduction on disease epidemiology. This report describes the method and its use in The Gambia and Senegal.

WHO-coordinated IB-VPD sentinel hospital surveillance for meningitis is established at large (usually pediatric) referral hospitals. A limitation of sentinel surveillance has been the inability to calculate meningitis incidence rates because of the absence of a known denominator of children at-risk for meningitis. In 2012, WHO, under the guidance of a technical advisory group, developed a method for estimating the denominator of at-risk children to calculate hospitalization rates for meningitis among children in the catchment area of a sentinel hospital (4). The method was finalized based on results of pilot tests conducted in IB-VPD surveillance network countries and is similar to a method recently developed for influenza sentinel hospital surveillance (Anthony Mounts, MD, Global Influenza Programme, WHO, personal communication, March 1, 2013).

Estimation of the denominator begins with determining whether population estimates for children aged <5 years are available by district* from census, civil registration, or other administrative data, and whether hospital admission log books or other hospital sources contain information on patient place of residence. If residence information is missing or can not be determined for a large number of cases, the method can not be applied. If these data are available, sentinel hospital records are reviewed to identify all children aged <5 years admitted with suspected meningitis during a recent 2–3 year period. For surveillance, WHO defines suspected meningitis as illness in a child aged <5 years with a clinical diagnosis of meningitis or with sudden onset of fever (>101.3°F [>38.5°C] rectal or >100.4°F [>38.0°C] axillary) and either neck stiffness, altered consciousness with no alternative diagnosis, or other meningeal signs (5). For this retrospective record review, an admission diagnosis of meningitis or an indication for lumbar puncture also met the surveillance case definition. Under the WHO method, hospital admission registers, specimen collection logbooks, laboratory records of CSF specimens received, and IB-VPD surveillance databases are reviewed for the specified time period. Individual patient charts are not reviewed. Patients aged <5 years with suspected meningitis are identified, and information on residence is abstracted. To produce reliable estimates, ≥100 suspected meningitis cases should be identified over a minimum of 2 (ideally consecutive) years.

Cases are spot-mapped by district of residence to visualize geographical dispersion and outliers. Districts are then rank-ordered according to the number of cases, and district-specific hospitalization rates for children aged <5 years are calculated using administrative population estimates. Using this rank-order, the sentinel hospital geographical catchment area is defined as those districts where 80% of suspected meningitis patients reside; the catchment area may be adjusted to fewer or more districts based on district-specific rates or expert local opinion. Expert national opinion is then sought to identify nonsentinel hospitals likely to admit at least 10% as many children aged <5 years with suspected meningitis as the sentinel hospital among children residing in the sentinel hospital’s geographical catchment area. These nonsentinel hospitals are visited and the number of children with suspected meningitis residing in the sentinel hospital catchment area is determined by reviewing clinical, laboratory, and residence data, using the same methods as at the sentinel hospital. The denominator of at-risk children is estimated by multiplying the population aged <5 years in the identified districts by the percentage of suspected meningitis patients aged <5 years who are admitted to sentinel hospitals. For example, if 90 of 100 patients among catchment area residents are admitted to the sentinel hospital and 10 cases are admitted to nonsentinel hospitals, the total at-risk population aged <5 years would be 90% of the children residing in the geographic catchment area. Annual meningitis hospitalization rates and 95% confidence intervals (CIs) were calculated using the mid-P exact test in OpenEpi software.

Pilot testing of the method was conducted in The Gambia and Senegal during 2012 to assess its feasibility. Before the assessment, Hib vaccines were introduced in the routine childhood immunization programs in both countries (Table), and one IB-VPD sentinel hospital in each country’s capital city conducted sentinel surveillance for meningitis among children aged <5 years. Pneumococcal conjugate vaccine was introduced in The Gambia 1 year before the data abstraction period (Table). In The Gambia and Senegal, catchment areas comprising five districts accounted for 87% and 79%, respectively, of suspected meningitis patients admitted to the sentinel hospital, with total populations aged <5 years of 130,794 and 300,842, respectively. Among catchment area residents aged <5 years, nonsentinel hospitals accounted for 13% of suspected meningitis admissions in The Gambia (one hospital) and 21% of suspected meningitis admissions in Senegal (two hospitals). The annual sentinel hospitalization rate for suspected meningitis was 42.8 per 100,000 population (CI = 34.9–52.0) in The Gambia and 119.8 (CI = 110.3–130.0) in Senegal.

Sentinel hospitals conducting IB-VPD surveillance need to maintain consistent practices, including case identification, specimen collection, and laboratory procedures, among a stable at-risk population of children to monitor meningitis trends over time. Time trends are used to assess changes in admissions for suspected meningitis, laboratory-confirmed infections, and serotype or strain of causative organisms after vaccine introductions. This report describes a method for estimating denominators of at-risk children to better understand sentinel hospital surveillance data. Use of this method is limited to geographic areas with existing administrative population estimates, large sentinel hospitals that admit ≥100 children aged <5 years with suspected meningitis over a 2-year period (a minimum of 2 years is required because the incidence of meningitis can vary substantially from year-to-year), and hospitals with easily accessed records that include residence information. Accuracy depends on quality of population estimates and hospital records. Ideally, the denominator should be reassessed every 2 years, because population migration and changes in health-care–seeking can occur over time.

Because most children with meningitis are treated as inpatients, with admissions generally limited to a small number of health facilities that have the capacity to care for severely ill patients, this method may also be used to estimate meningitis incidence. However, the hospitalization rate might underestimate or overestimate actual meningitis incidence rates because children with meningitis might die at home, and some might be misdiagnosed on admission or diagnosed differently at sentinel versus nonsentinel hospitals. Thus, rates derived using this method should be interpreted cautiously; in developing countries with poor population access to health care, these estimated incidence rates likely represent minimum estimates. In sentinel hospitals with reliable and consistent laboratory diagnostic practices, IB-VPD surveillance can be used to estimate hospitalization rates for probable bacterial meningitis† as well as laboratory-confirmed Hib or pneumococcal meningitis. Such hospitals with at least 2 years of pre-PCV introduction data can assess trends in the rates of laboratory-confirmed pneumococcal meningitis before and after vaccine introduction. Comparison with Hib and pneumococcal meningitis incidence estimates for children aged <5 years from WHO’s Global Burden of Disease project§ or rigorous epidemiologic studies can help assess the validity of incidence rates calculated using this rapid assessment method.

WHO provides support to countries to strengthen national capacity to better monitor and evaluate vaccination programs by using cased-based surveillance with laboratory confirmation, a goal of the Global Vaccine Action Plan.¶ During 2012, a total of 57 countries reported meningitis sentinel surveillance data to the WHO-coordinated IB-VPD surveillance network. The ability to calculate rates of hospitalization for suspected meningitis strengthens the global IB-VPD surveillance network’s ability to compare data among sites, over time, and with special studies (e.g., vaccine impact evaluations).

## Figures and Tables

**TABLE:** Results of pilot testing to estimate the annual hospitalization rate for suspected meningitis at sentinel hospitals participating in the World Health Organization (WHO)–coordinated invasive bacterial vaccine preventable diseases surveillance network during 2010–2011 — The Gambia and Senegal, 2012

Characteristic	The Gambia	Senegal
**Year vaccine introduced**		
Hib3	1997	2005
PCV	2009	N/A
**Estimated 2011 coverage for children aged <12 months (%)** *		
Hib3	(96)	(83)
PCV	(93)	N/A
**Suspected meningitis admissions among children aged <5 years from the geographic catchment area**		
Admitted to sentinel hospital†	97	567
Admitted to nonsentinel hospitals	15	154
**Population of children aged <5 years in geographic catchment area**		
Annual total	130,794	300,842
Annual total adjusted for nonsentinel admissions	113,277	236,584
Population adjusted for 2-year analysis period	226,554	473,169
Annual hospitalization rate for suspected meningitis (95% confidence interval)§	42.8 (34.9–52.0)	119.8 (110.3–130.0)

**Abbreviations:** Hib3 = third dose of *Haemophilus influenzae* type b vaccine; PCV = pneumococcal conjugate vaccine; N/A = not available.

* Coverage based on joint WHO and United Nations Children’s Fund (UNICEF) estimate, Additional information available at http://apps.who.int/immunization_monitoring/globalsummary/timeseries/tscoveragehib3.html.

† Sentinel hospitals: Royal Victoria Teaching Hospital, Banjul, The Gambia; Albert Royer Hospital, Dakar, Senegal.

§ Per 100,000 children aged <5 years residing in the geographic catchment area during the period of record abstraction, adjusted for nonsentinel hospital utilization.

## Footnotes

* Smallest administrative unit for which population estimates are available.

† WHO defines probable bacterial meningitis as a suspected meningitis case with CSF examination showing at least one of the following: 1) turbid appearance, 2) leukocytosis (>100 cells/mm^3^), or 3) leukocytosis (10–100 cells/mm^3^) and either an elevated protein (>100 mg/dL) or decreased glucose (<40 mg/dL). If protein and glucose results are not available, diagnosis is made using the first two conditions (i.e., turbid appearance or leukocytosis [>100 cells/mm^3^]).

§ Additional information available at http://www.who.int/immunization_monitoring/burden/Pneumo_hib_estimates/en/index2.html.

¶ Additional information available at http://www.who.int/immunization/global_vaccine_action_plan/en.
